# Impact of calcium hydroxide as an intracanal medicament on postoperative pain in diabetic and non-diabetic patients with symptomatic apical periodontitis and pulp necrosis – a double-blind randomized controlled clinical trial

**DOI:** 10.4317/jced.63428

**Published:** 2025-12-30

**Authors:** Alka Gurawa, Alpa Gupta, Jasmine Jaideep Rayapudi, Vivek Aggarwal, Shreya Asija

**Affiliations:** 1 MDS. Department of Conservative Dentistry and Endodontics, School of Dental Sciences, Manav Rachna Dental College, MRIIRS, Faridabad, Haryana, India; 2 MDS, PGDHPE. Department of Conservative Dentistry and Endodontics, School of Dental Sciences, Manav Rachna Dental College, MRIIRS, Faridabad, Haryana, India; 3 MDS. Jamia Millia Islamia, Ghaffar Manzil Colony, Jamia Nagar, Okhla, New Delhi; 4 BDS. Department of Conservative Dentistry and Endodontics, School of Dental Sciences, Manav Rachna Dental College, MRIIRS, Faridabad, Haryana, India

## Abstract

**Background:**

This study aimed to evaluate the effect of calcium hydroxide Ca(OH)2 as an intracanal medicament on postoperative pain in diabetic and non-diabetic patients with symptomatic apical periodontitis and pulp necrosis.

**Material and Methods:**

A double-blind, prospective randomized controlled clinical trial was conducted on 96 patients (48 diabetics and 48 non-diabetics) aged 30-65 years. Patients were allocated into four groups: Group A (non-diabetic with Ca(OH)2), Group B (diabetic with Ca(OH)2), Group C (non-diabetic without Ca(OH)2), and Group D (diabetic without Ca(OH)2). Endodontic treatment was performed, and postoperative pain was assessed using the Heft Parker Visual Analog Scale (HPVAS) at 6, 24, 48, 72, 96, 120, 144, and 168 hours. Statistical analysis included Kruskal-Wallis, Dunn's post hoc, and Kaplan-Meier survival tests.

**Results:**

At baseline and 6 hours, pain scores were comparable across groups (p &gt; 0.05). From 24 to 120 hours, significant differences were observed (p &lt; 0.05), with Group A showing the fastest and most consistent pain reduction, while Group D reported the highest pain levels. Groups B and C demonstrated intermediate relief with comparable trends. By 168 hours, all groups achieved near-zero pain levels. Kaplan-Meier analysis confirmed the earliest median pain relief in Group A (72 hours), delayed recovery in Groups B and C (120 hours), and the slowest in Group D.

**Conclusions:**

Calcium hydroxide significantly reduced postoperative pain, with the greatest benefit in non-diabetic patients. Its effect was more decisive than diabetes status, underscoring its clinical significance as a reliable intracanal medicament for managing postoperative pain in both healthy and systemically compromised patients.

## Introduction

Diabetes Mellitus (DM) is a systemic metabolic disorder with immunosuppressive characteristics, characterized by elevated glycosylated haemoglobin levels (&gt;6.5), as defined by the American Diabetes Association (1). Its global occurrence was 171 million in 2000 and is expected to rise to 366 million by 2030 (2). As a pan-systemic disease, DM adversely affects multiple organ systems. In endodontics, diabetic patients presenting with Symptomatic Apical Periodontitis (SAP) are particularly challenging to treat due to delayed healing, often manifested as persistent pain. Fouad et al. reported a higher incidence of preoperative pain in diabetic cases (3), and previous studies have confirmed increased rates of postoperative pain and flare-ups in SAP patients (4 , 5). SAP is an inflammatory condition of the periradicular tissues caused by polymicrobial flora, which activates the nonspecific immune system. This innate immune response plays a critical role in periapical healing (6). Evidence indicates that DM acts as a disease modifier in apical periodontitis, as diabetic patients harbour more virulent and pathogenic microbial flora. Endodontic procedures such as biomechanical preparation and obturation may exacerbate this condition through over-instrumentation or apical extrusion of microbial and chemical agents, thereby intensifying postoperative pain (7). Konagala et al. attributed post-endodontic pain to inflammatory mediators, particularly prostaglandins, which are key contributors to pulpal and periradicular disease (8). Furthermore, residual or extruded microbes can precipitate acute apical periodontitis, worsening pain outcomes (5). The standard approach to managing such cases involves thorough debridement of the root canal system, followed by the placement of a suitable intracanal medicament. Calcium hydroxide is the most commonly used intracanal medicament, supported by extensive literature demonstrating its antimicrobial and anti-inflammatory efficacy. While several studies have investigated its effectiveness compared with other medicaments or different vehicles, and some have examined periapical healing in diabetic patients, very few have assessed its role specifically in terms of microbiological outcomes or subjective postoperative pain in this population. To our knowledge, no study has directly compared the efficacy of calcium hydroxide as an intracanal medicament in diabetic versus non-diabetic patients till date. Therefore, this randomized controlled trial was designed to generate evidence-based insights on the effect of calcium hydroxide as an intracanal medicament on postoperative pain in diabetic and non-diabetic patients with pulp necrosis and symptomatic apical periodontitis.

## Material and Methods

**Figure 1:**
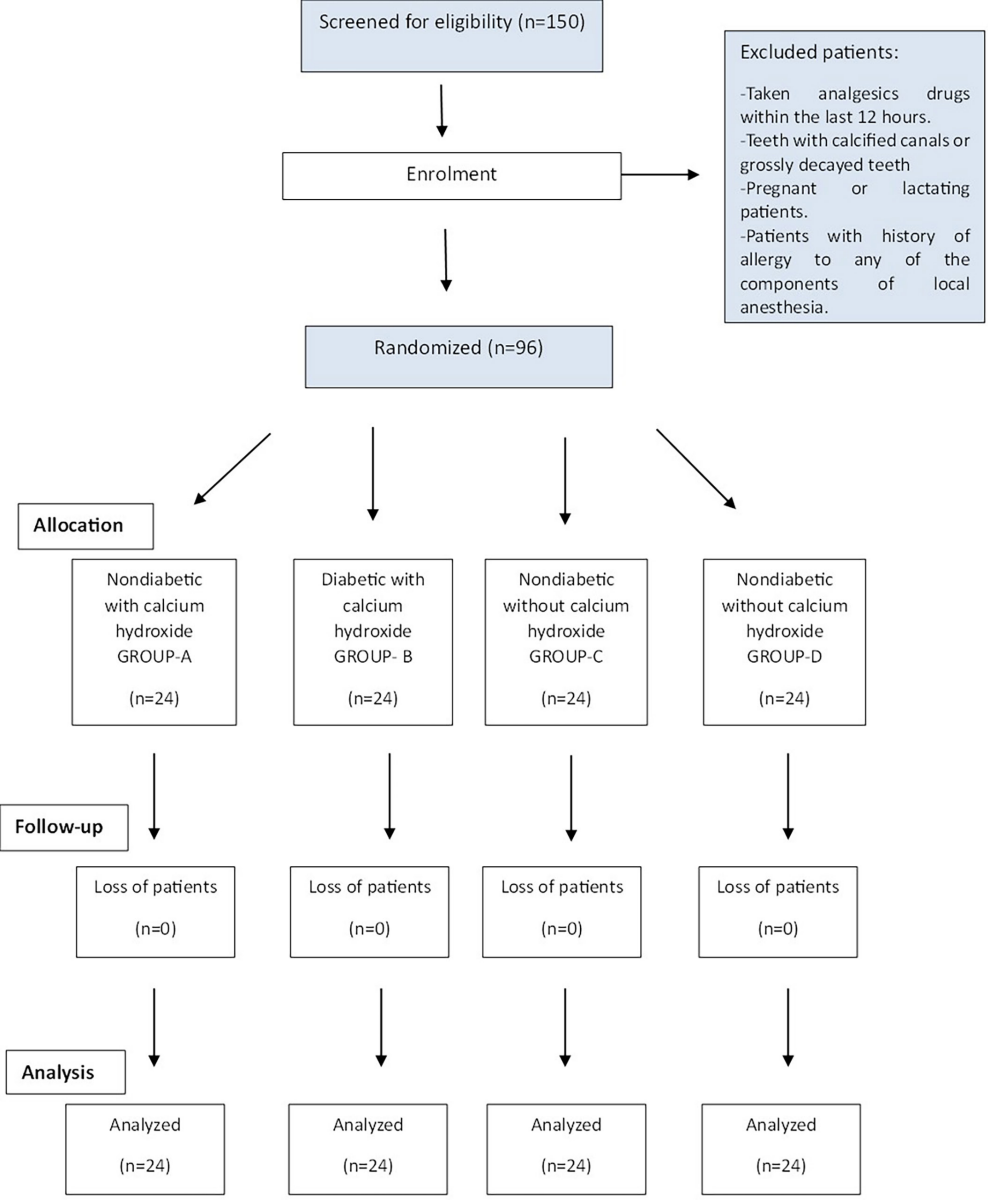
CONSORT flowchart of the study.

- Study design, trial registration, and ethical approval This double-blind, prospective randomized control trial was performed in the Department of Conservative Dentistry and Endodontics at Manav Rachna Dental College, Faridabad, Haryana, India. Ethical approval was taken from the Institutional Review Board (Ethics Committee) of Manav Rachna Dental College (Ref. No: MRDC/IEC/2019/530). The study protocol was registered in the Clinical Trials Registry of India (www.ctri.icmr.org.in) under the identifier number CTRI/2020/02/023370. A Consolidated Standards of Reporting Trials (CONSORT, 2010) flow diagram is shown in Figure 1.

1

- Sample size calculation The power analysis of the study was performed based on the previous study (9) and minimal clinically important differences in the Heft Parker Visual Analog Scale (HPVAS) scores. The sample size calculation showed that a minimum of 24 patients would be needed in each intervention arm to detect any significant difference in pain level, with alpha, beta errors of 0.05 and 0.20 respectively and adjusting the power of the study to 80%. - Recruitment and Eligibility criteria The study recruited patients between March 2020 and December 2021, with follow-up conducted for up to 7 days after the first visit. Following comprehensive clinical and radiographic examinations, 150 subjects aged 30-65 years were initially enrolled in the study. Only patients with symptomatic mandibular posterior teeth with pulp necrosis and radiographic evidence of apical periodontitis were included (according to the classification of pulpal and periapical diseases proposed by the AAE and ESE). Exclusion factors were patients, who had taken painkillers or anti-inflammatory drugs within the last 12 hours, patients with symptomatic teeth showing unfavorable root morphology (open apex, severely curved, dilacerated, external or internal root resorption, severely sclerosed, and obliterated) and patients with occlusal interferences involving the offending tooth. Ultimately, 96 patients meeting the inclusion and exclusion criteria were enrolled in the study. To establish the metabolic control status of diabetic patients, HbA1C was registered on the day of treatment initiation. Acceptable glycemic control was defined according to the American Association of Clinical Endocrinologists as HbA1C (1). - Randomization and Blinding The subjects for the clinical trial were divided into 2 groups: type 2 diabetic patients (48 patients) and non-diabetic patients (48 patients). Within each group the participants were further divided into two distinct groups to evaluate the outcomes- Non-Diabetic Patients Group A: Non-diabetic patients receiving calcium hydroxide intracanal medicament. Group C: Non-diabetic patients who do not receive any intracanal medicament. Diabetic Patients Group B: Diabetic patients receiving calcium hydroxide intracanal medicament. Group D: Diabetic patients who do not receive any intracanal medicament. (Supplement 1) (http://www.medicinaoral.com/medoralfree01/aop/jced_63428_s01 A researcher who had not contributed in the study generated the randomization sequence using a computer random table generator (www.random.org) with a 1:1 allocation ratio. Randomization was done within diabetic group and non-diabetic group for the two intracanal medicament groups (with and without). The initial clinical examination included checking for pain, fistula, oedema, and tenderness. The teeth that did not respond to electric pulp testing (Electron Pulp Tester, DIGITEST, Parkell, New York)) and cold sensibility test (Roeko ENDO-FROST cold spray; Coltene, Switzerland) were identified as pulp necrosed. The healthy contralateral tooth was used as a control for sensibility and percussion tests. Patients were diagnosed in the outpatient unit of the Department of Conservative Dentistry and Endodontics whether he/she is diabetic or non-diabetic by Random Blood Sugar test (&gt; 200 mg/dl) using a glucometer (Gluco-one by Dr Morepen). A confirmatory test for diabetes was done by fasting blood sugar (&gt; 126 mg/dl) and HbA1C test (&gt; 6.5). Diabetic patients were advised to continue with their medical treatment of DM (oral hypoglycemic agents), diet, and lifestyle without alterations during the study period. The enrolled patients were explained the objectives, the procedure involved, follow up pain evaluation and potential discomfort after which they were requested to sign the pre-approved informed consent form. The participants and non-participating researchers analyzing the results were blinded to the intervention. The allocation concealment was done by placing the sequence in a sealed opaque envelope, which was then given to the dental assistant. According to the randomized sequence, the eligible patients were given serial numbers and allotted to the groups by the non-participating researcher. Detailed records of the sequence and serial numbers were maintained to ensure the success of the blinding procedure. - Standardized Endodontic Treatment Protocol The treatment was completed in a two-visit approach using a standardized treatment protocol. The selected tooth was anaesthetized (with 2% lidocaine, 1.8mL with 1:100,000 epinephrine) using the inferior alveolar nerve block technique and isolated with a rubber dam. The standard treatment procedure was as follows for all the groups: access cavity was prepared using sterile carbide burs followed by access cavity refinement with Endo Z bur. The glide path was attained with the help of an ISO stainless steel hand K-file #10 number (Mani, Inc. Japan). The pulp chamber was debrided, and the working length was established with the aid of the electronic apex locator (Root ZX; J Morita, Tokyo, Japan) using #15 K files. Rotary instrumentation for all the teeth in each group was performed with the TruNatomy file (Dentsply, India). Mesial canals were instrumented up to apical preparation of size 26 and 0.04 taper. The distal canal was prepared up to size 35 and 0.04 taper accompanied by copious irrigation between each file change (10). Irrigation was performed with 5ml 3% sodium hypochlorite, 5ml 17% EDTA for 1 minute followed by 5ml 3 % sodium hypochlorite using a 30-G endodontic needle (Maxi-Probe, Dentsply Maillefer) 2mm short of the apex. Calcium hydroxide powder was mixed with sterile saline in the ratio of 1.5:1 (wt./vol) to attain a paste-like texture. The canals were dried with absorbent paper points. The calcium hydroxide paste was placed in the canal with the help of lentulospirals (Mani Inc, Tachigiken, Japan) rotating at slow speed, approximately 1 mm short of the working length in Group A and Group-B (11). No intracanal medicament was placed in the canals of patients allocated in Group C and Group- D. A Teflon pellet was placed over each canal orifice, and the cavity was filled with temporary cement (MD Temp Plus, META BIOMED, Germany). The patients were informed about the chances of occurrence of pain and suitable analgesics were also prescribed after verifying any possible drug allergy. The HPVAS was explained to the patients, and they were asked to record the intensity of pain on HPS after 6 hours, 24 hours (day 1), 48 hours (day 2) for 7 days after the first visit. HPVAS consisted of a 170-mm line with 0 indicating the absence of pain and 170 indicating the worst pain possible (12). Patients were also further instructed to take an analgesic (400 mg ibuprofen) (10). In case of persistent pain, the patient was advised to inform the clinician by calling/texting and reporting to the department. Patients were also instructed to record the intensity of pain and indicate whether they had taken pain medication (Yes/No). After one week HPVAS scales were collected, recorded and tallied with the record that was being maintained as per verbal communication through phone at the prescribed time intervals. After thorough confirmation of the absence of any signs or symptoms that may indicate a persistent infection, the teeth were isolated again under a rubber dam. The temporary filling was carefully removed from the walls of the tooth. The pulp chamber was debrided of any temporary filling and a similar irrigation protocol of 17% EDTA and 3% NaOCl was followed to remove any trace of intracanal medicament in the groups where calcium hydroxide was used. Canals were dried with sterile paper points and obturated using the lateral compaction technique with resin based AH Plus sealer (Dentsply DeTrey, Konstanz, Germany). Coronal seal was placed with composite restoration followed by checking for any occlusal interference. Digital periapical radiography was taken to review the final quality of the restoration. Patients who experienced pain were instructed to take an analgesic (400 mg ibuprofen). It is noteworthy to mention here that there was no attrition reported as detailed verbal communication was maintained with each patient from each group. - Data Collection The independent variables were sex (female or male), age (in years), preparation time in minutes, periapical condition (without or with lesion), HbA1C status of the patients and placement of intracanal medicament. The dependent variables were postoperative pain at 6, 24, 48, and 72 hours followed by consequently 4 more days, and the intake of postoperative pain medication (Yes/No) were recorded. The Heft Parker Visual Analog Scale (HPVAS) was explained to the patients, and they were asked to record the intensity of pain after 6 hours, 24 hours (day 1), 48 hours (day 2), for 7 days after the first visit. - Statistical analysis Statistical analysis was performed at a 5% significance level. Data preprocessing, including cleaning, reduction, and integration, was conducted prior to analysis. Assumptions of normality were assessed using Shapiro-Wilk tests and QQ plots. Descriptive statistics were reported as mean ± standard deviation. For inferential analysis, Mann-Whitney U tests, Kruskal-Wallis tests, and post hoc Dunn tests were applied to evaluate significance and pairwise comparisons. Survival outcomes were analyzed using Kaplan-Meier curves and multivariate log-rank tests. Categorical variables were assessed using chi-square tests, and exploratory data analysis, including bar charts, was performed to visualize trends.

## Results

were interpreted in the context of treatment efficiency and variable relationships. Analyses were conducted using Excel, Power BI, and Python 3.11.4 with Pandas, Lifelines, NumPy, and SciPy libraries. Results - The patients' return rate was 100% with all groups. The results of the descriptive analysis of demographic data are shown in Table 1.

Table 1

**Figure 2:**
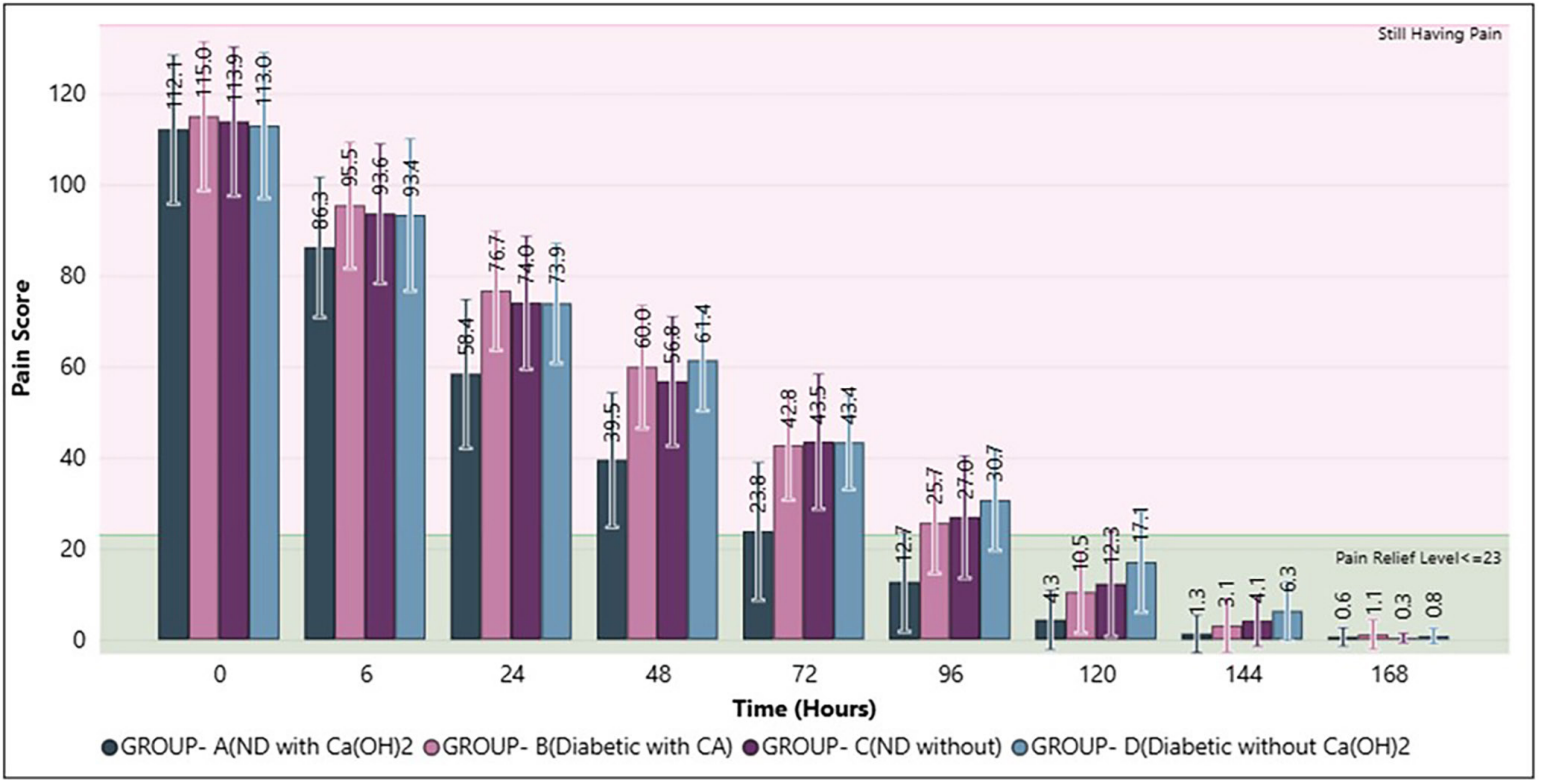
Comparison of Postoperative Pain Reduction across Groups over Time.

The study group consisted of 96 participants, including 51 women and 45 men. The Chi-square test for gender distribution (p = 0.9276) showed no significant difference across the four groups, indicating comparable proportions of males and females. Similarly, the Kruskal-Wallis test for age (p = 0.4287) revealed no significant difference, suggesting that the groups were age-matched. The comparison of pain scores (Mean ± SD) across groups over time revealed that, while gradual pain reduction was observed in all groups, Ca(OH)2 proved most effective, especially in non-diabetic patients, though intergroup differences diminished by the end of the follow-up period (Table 2, Fig. 2).

Table 2

2

The Post Hoc Dunn Test for pair-wise group comparison of pain scores over time demonstrated that, that Group A consistently achieved the fastest and most effective pain reduction, whereas Group D (diabetic without Ca(OH)2) demonstrated the slowest recovery. The remaining groups showed comparable pain relief patterns, with differences diminishing at later time points (Table 3).

Table 3

The Kruskal-Wallis test (p &lt; 0.05 at all-time points) indicated statistically significant differences in pain reduction patterns among the groups. Group A (non-diabetic with Ca(OH)2) showed the fastest and most consistent decline in pain scores, reaching near-zero levels by 120-144 hours. In contrast, Group D (diabetic without Ca(OH)2) consistently recorded the highest pain scores across time, showing the slowest recovery. By 168 hours and at the postoperative evaluation, pain scores in all groups had reduced to minimal values, although differences in the early and mid-phase confirmed the superior effect of Ca(OH)2, particularly in non-diabetic patients. Time analysis using quartiles and Multivariate-Log-Rank test showed the minimum time required to achieve pain relief (Table 4).

Table 4

**Figure 3:**
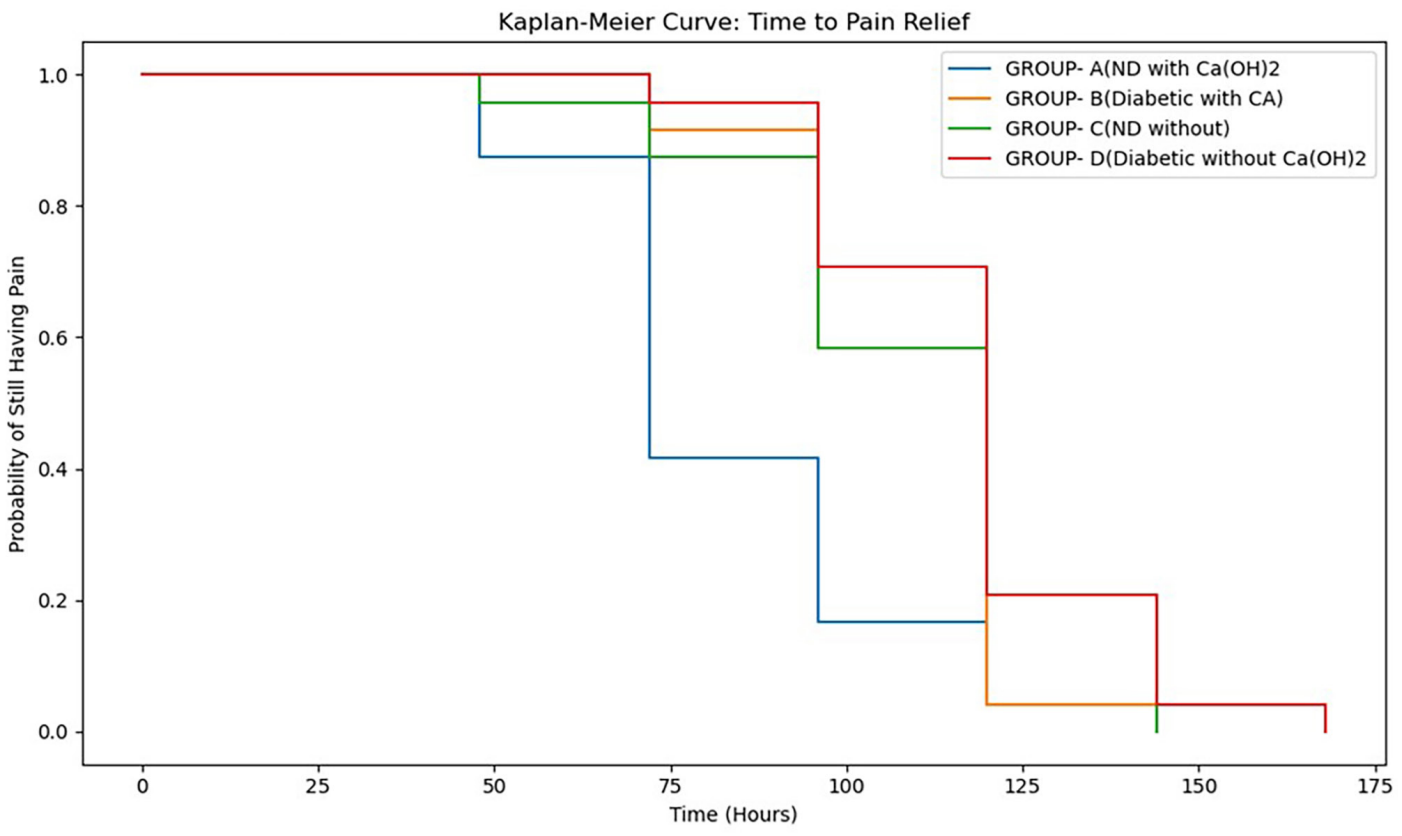
Kaplan–Meier Analysis of Time to Pain Relief (Event: Pain Score ≤ 23).

The Kaplan-Meier survival analysis (event = pain relief 23) demonstrated significant differences in the time to pain relief among groups (Multivariate Log-Rank test, p = 0.000013), (Fig. 3) Group A (non-diabetic with Ca(OH)2) achieved pain relief earliest, (median 72 hours), delayed relief in Groups B and C (median 120 hours), and the slowest recovery in Group D.

3

## Discussion

This double-blind randomized control trial was carried out to clinically assess the efficacy of calcium hydroxide as an intracanal medicament in diabetic and non-diabetic patients with symptomatic apical periodontitis and pulp necrosis using the Heft Parker Visual Analog Scale (HPVAS) (12). We attempted to reduce confounding factors by strict adherence to inclusion and exclusion criteria. Mandibular posterior teeth were included in the study as they are known for complex anatomy with most of the bifurcations and trifurcations at the apex which make it difficult to clean and shape (13 , 14). Mandibular posterior teeth also have shown an increased predisposition to SAP. Hence patients with SAP and pulp necrosis in mandibular posterior teeth were enrolled for the study. Research demonstrates that while chemo-mechanical preparation significantly reduces bacterial load, residual microorganisms persist in dentinal tubules and anatomical complexities of the root canal system. This justifies the placement of intracanal medicaments, particularly calcium hydroxide, as a two-visit protocol which provides sustained antimicrobial activity that continues the disinfection process over an extended period (15). Symptomatic apical periodontitis cases have a higher concentration of molecular mediators of inflammation. The increased concentration of inflammatory mediators causes a decreased firing threshold of pain thus enabling previous noxious or non-noxious stimuli (e.g. introducing endodontic instruments, irrigants or materials) to aggravate the level of pain (16). In this study, diabetic patients without calcium hydroxide took the maximum number of pain medications and had the highest mean pain scores at all time points evaluated compared to the other groups. This can be probably attributed to the presence of more anaerobic bacteria in the diabetic group which can enhance the inflammatory response in the periapical area and can aggravate the postoperative pain (17). The immunosuppressive role of DM contributes to delayed healing. Ahmed et al. (2013) reported that the presence of MMP-9 was more in symptomatic apical periodontitis cases (18). The matrix metalloprotein released from macrophages MMP-9 up-regulates the level of prostaglandin (PGE2) leading to bone resorption and pain. Sarmento et al 2020 evaluated the immunoexpression of MMP-9 in apical periodontitis lesions of normoglycemic and diabetic individuals and concluded that quantitatively, diabetics had a higher percentage of strong MMP-9 expression than normoglycaemics (19). The altered immune response increased anaerobic bacteria colonies and increased expression of MMPs may increase the incidence of postoperative pain and poor periapical healing in diabetic patients. Statistical differences between groups were observed only during the early postoperative period, with no significant differences beyond the initial hours or days. Across all groups, pain scores decreased steadily over time, with the most pronounced reduction observed in Group A (non-diabetic with Ca(OH)2), which reached the pain-relief threshold earliest. Groups without Ca(OH)2 (C and D) and the diabetic groups (B and D) showed slower pain reduction, with Group D (diabetic without Ca(OH)2) maintaining the highest pain levels throughout. By 144-168 hours, pain scores in all groups approached minimal levels, indicating gradual and consistent postoperative pain relief, but with clear benefits of Ca(OH)2, especially in non-diabetic patients. The inclusion of the non-diabetic groups with (Group A) and without calcium hydroxide (Group C) have shown significantly lower mean scores at all time points evaluated and have enabled a clear comparison to the diabetic groups. This further strengthens the fact that Diabetes Mellitus act as an immunomodulator and thus results in increased post-operative pain and hence justifies our decision to include non-diabetics in the present randomized control trial. Another noteworthy finding that was proved in our study was the increase in postoperative pain in the diabetic groups (Group B and Group D) after root canal instrumentation which suggests a possible flare up thus supporting the finding by Torabinejad in 1994 (16). The polymicrobial flora can be minimized after the placement of a suitable intracanal medicament after thorough chemo-mechanical preparation. Calcium hydroxide is the most widely used intracanal medicament. Its high pH modifies the biological properties of bacterial lipopolysaccharide in the cell walls of gram-negative species, thus inactivating the membrane transport mechanisms. The release of hydroxyl ions in an aqueous setting is responsible for the antimicrobial activity of calcium hydroxide. Their lethal effects on bacterial cells are triggered by damage to the bacterial cytoplasmic membrane, protein denaturation, and damage to DNA. Madhubala et al. 2011 and Almyroudi et al. 2002 reported that Calcium hydroxide shows significant reduction in amount of E faecalis colonies than control group at 1, 2, 3, 7,8,14 days (11 , 20). Bedran et al. 2021 concluded that calcium hydroxide reduces endotoxin level when used as an intracanal medicament (21). Calcium hydroxide also reduces the level of MMP-9 (22 , 23 , 24). In this way by decreasing anaerobic bacteria colonies, endotoxin and MMP-9, calcium hydroxide can reduce the inflammation in periapical area which further can minimize postoperative pain. Numerous studies have evaluated the role of various vehicles with calcium hydroxide since the vehicle employed with calcium hydroxide paste influences the diffusion capacity of calcium hydroxide. Zmener et al. 2007 and GG Mori et al. 2009 concluded that paste of calcium hydroxide with distilled water released hydroxyl ions more rapidly on the 1st day (25 , 26). This is evident in our study where the post-operative pain was significantly reduced at 24h in both Group A and B where calcium hydroxide paste was prepared with distilled water and placed in the canals with the help of lentulospirals as an ICM in both diabetic and non-diabetic patients (27). The clinical efficacy of a medicament can be assessed only by evaluating the pain scale. Hence the inter appointment pain was measured and compared using the Heft Parker Scale Visual Analogue (HPVAS) in diabetic and non-diabetic individuals with symptomatic apical periodontitis after placement of calcium hydroxide. The pain evaluation was done for up to one week to monitor the pain intensity. The findings indicate that calcium hydroxide markedly improves early postoperative pain control in patients with symptomatic apical periodontitis, with the greatest benefit observed in non-diabetic individuals, while diabetic patients without intracanal medicament experienced the slowest recovery. Importantly, the presence of calcium hydroxide had a stronger impact on pain outcomes than diabetes status alone, as demonstrated by comparable recovery patterns between diabetic patients with calcium hydroxide and non-diabetic patients without it. These results suggest that the antimicrobial and anti-inflammatory properties of calcium hydroxide-such as its ability to neutralize bacterial endotoxins, reduce anaerobic microbial load, and downregulate inflammatory mediators like MMP-9 and prostaglandins (10 , 17 - 20) can effectively counteract the heightened inflammatory response and delayed healing typically associated with diabetes (16 , 18). This reinforces its clinical value as a routine intracanal medicament in both healthy and medically compromised patients. A limitation of this study is that only calcium hydroxide was evaluated as an intracanal medicament, although its efficacy is known to improve when combined with agents such as chlorhexidine (28). Restricting the analysis to calcium hydroxide alone may not fully represent the potential of alternative or combination regimens. Future studies should compare calcium hydroxide with other medicaments and combination protocols in both diabetic and non-diabetic patients to identify the most effective strategy for reducing postoperative pain and enhancing periapical healing. This study is also limited by the inherent challenge of completely removing calcium hydroxide intracanal medicament from the root canal system prior to obturation, as residual material may persist despite current irrigation and instrumentation techniques, potentially affecting sealer adaptation and long-term treatment outcomes. Future research should aim to evaluate and optimize novel removal protocols and technologies, as well as assess the clinical impact of residual calcium hydroxide on the quality of obturation and long-term prognosis in diverse and complex root canal anatomies.

## Conclusions

This double-blind randomized controlled trial proved that calcium hydroxide significantly reduced postoperative pain in patients with symptomatic apical periodontitis and pulp necrosis. Non-diabetic patients receiving calcium hydroxide achieved the most rapid and consistent pain relief, while diabetic patients also benefited, though with comparatively slower recovery. Groups without intracanal medicament consistently showed delayed healing, with diabetic patients without calcium hydroxide reporting the highest pain scores. As a clinical significance, this study highlights the beneficial effect of calcium hydroxide in reducing early postoperative pain following endodontic treatment. This early analgesic effect is attributed to its antimicrobial and anti-inflammatory properties, as calcium hydroxide neutralizes bacterial endotoxins, reduces periapical inflammation, and creates an alkaline environment that suppresses inflammatory mediator release. Clinically, this translates to greater patient comfort, lower analgesic intake, and fewer flare-ups during the interappointment period, reinforcing the importance of its use in symptomatic apical periodontitis and pulp necrosis cases for pain control and improved healing outcomes. Calcium hydroxide remains a cost-effective and reliable option, providing superior outcomes in non-diabetic patients and meaningful benefits even in diabetic individuals, who are at greater risk of persistent inflammation and delayed healing. Its routine use can improve patient comfort, satisfaction, and overall treatment success in endodontic practice.

## Figures and Tables

**Table 1:** Baseline Comparison of Demographic Characteristics across Study Groups.

Groups	GROUP- A(ND with Ca(OH)2	GROUP- B(Diabetic with CA)	GROUP- C(ND without)	GROUP- D(Diabetic without Ca(OH)2	P values
Gender	Chi-Square test				0.9276
F	14	12	13	12	
M	10	12	11	12	
Age	Kruskal-Wallis test				0.4287
	49.62 ± 8.11	52.92 ± 6.51	51.08 ± 7.52	50.67 ± 6.07	

1

**Table 2:** Comparison of Pain Scores (Mean ± SD) Across Groups Over Time.

Time Periods	GROUP- A(ND with Ca(OH)2	GROUP- B(Diabetic with CA)	GROUP- C(ND without)	GROUP- D(Diabetic without Ca(OH)2	Kruskal-Wallis-test p values
Preop top	114.21 ± 16.27	116.17 ± 16.52	115.25 ± 16.78	114.58 ± 16.32	0.9763
Preop top	114.21 ± 16.27	116.17 ± 16.52	115.25 ± 16.78	114.58 ± 16.32	0.9763
PREOP	112.12 ± 16.72	115.04 ± 16.70	113.88 ± 16.71	113.00 ± 16.34	0.9173
6 HR	86.25 ± 15.73	95.46 ± 14.17	93.62 ± 15.68	93.38 ± 17.10	0.2442
24 HR	58.42 ± 16.71	76.71 ± 13.41	74.04 ± 14.94	73.92 ± 13.47	0.0013*
48 HR	39.50 ± 15.09	59.96 ± 13.83	56.79 ± 14.52	61.42 ± 11.34	0.0000*
72 HR	23.83 ± 15.48	42.75 ± 12.30	43.54 ± 15.17	43.38 ± 10.56	0.0000*
96 HR	12.67 ± 11.15	25.67 ± 11.34	26.96 ± 13.74	30.71 ± 11.32	0.0000*
120 HR	4.33 ± 6.68	10.46 ± 9.17	12.25 ± 11.82	17.08 ± 11.27	0.0001*
144 HR	1.29 ± 4.20	3.12 ± 6.02	4.12 ± 5.70	6.29 ± 6.66	0.0066*
168 HR	0.58 ± 2.08	1.12 ± 3.33	0.33 ± 1.09	0.79 ± 1.74	0.8132
Postop top	1.17 ± 2.48	0.83 ± 1.93	0.96 ± 1.85	0.29 ± 1.08	0.4801

2

**Table 3:** Pairwise Group Comparisons of Pain Scores Over Time (Post Hoc Dunn Test).

Pairwise Group Comparisons using Post Hoc Dunn Test	Time Periods					
	24 HR	48 HR	72 HR	96 HR	120 HR	144 HR
GROUP- A(ND with Ca(OH)2 vs GROUP- B(Diabetic with CA)	0.0003*	0*	0.0001*	0.0009*	0.0173*	0.0902
GROUP- A(ND with Ca(OH)2 vs GROUP- C(ND without)	0.0023*	0.00002*	0*	0.0004*	0.0089*	0.0426*
GROUP- A(ND with Ca(OH)2 vs GROUP- D(Diabetic without Ca(OH)2	0.0035*	0*	0*	0*	0*	0.0005*
GROUP- B(Diabetic with CA) vs GROUP- C(ND without)	0.5879	0.7322	0.8115	0.8377	0.8144	0.7386
GROUP- B(Diabetic with CA) vs GROUP- D(Diabetic without Ca(OH)2	0.4986	0.6223	0.9401	0.194	0.0344*	0.074
GROUP- C(ND without) vs GROUP- D(Diabetic without Ca(OH)2	0.8928	0.4039	0.8703	0.2739	0.0601	0.1463

3

**Table 4:** Time analysis using quartiles and Multivariate-Log-Rank test.

Groups	Quartile Show the minimum time (hours) to achieve Pain relief (<=23)			Multivariate-Log-Rank test p value
	Q1	Q2	Q3	
GROUP- A(ND with Ca(OH)2	72	72	96	0.000013*
GROUP- B(Diabetic with CA)	96	120	120	
GROUP- C(ND without)	96	120	120	
GROUP- D(Diabetic without Ca(OH)2	96	120	120	

4

## Data Availability

All the pertaining data are with the corresponding author and can be made available with a requisition mail to alpa.sds@mrei.ac.in
